# How to measure multidimensional quality of life of persons with disabilities in public policies - a case of Poland

**DOI:** 10.1186/s13690-022-00981-5

**Published:** 2022-11-10

**Authors:** Izabela Grabowska, Radosław Antczak, Jan Zwierzchowski, Tomasz Panek

**Affiliations:** SGH Warsaw School of Economics, Institute of Statistics and Demography, Warsaw, Poland

**Keywords:** Quality of life, Disability, Life quality domains, MIMIC

## Abstract

**Background:**

The aim of this paper is to construct a tool that can be used to measure multidimensional quality of life of persons with disabilities in comparison with population without disabilities for the purpose of monitoring of the UN Convention on the Rights of Persons with Disabilities (UNCRPD) in Poland.

**Methods:**

The Sen’s capability approach was applied to conceptualize the quality of life in various life domains. We followed guidelines of The Quality of Life Framework developed within the European Statistical System on choosing the life domains in which the QoL should be measured. The QoL scores in each domain (covered by the UNCRPD) were constructed using multiply indicators and multiple causes model (MIMIC). All analysis were based on 2018 EU-SILC data for Poland. We constructed quality of life indicators for population with and without disabilities and compared the differences.

**Results:**

Persons without disability experienced higher QoL as compared to population with disabilities, overall and in various domains. Lower average QoL of persons with disabilities is a result of a lower share of those who experience high QoL. The biggest difference is observed for health and for productive and main activity domains. For material conditions and economic security and physical safety there was a moderate difference recorded. For the leisure and social relations domain there is almost no difference observed. Additionally, we identified diversified associations between such factors as age, gender, household situation, education, partner status, urbanization, health on the QoL across domains and analysed populations.

**Conclusions:**

A tool developed in this paper can be calibrated to enable cross-country and in time comparisons between different populations and support evidenced-based social policy.

**Supplementary Information:**

The online version contains supplementary material available at 10.1186/s13690-022-00981-5.

## Introduction

Persons with disabilities are getting worldwide attention yet quantifying this population is still a challenge. Although international bodies introduce disability as a separate topic, developing programmes such as United Nations Disability Statistics Programme or International Disability Alliance (part of world Bank Group), still the coherent data on disability is hardly available. This is mainly due to various operationalization of disability term, therefore estimating total number of persons with disabilities as well as making international comparison is very challenging [1]. Global statistical databases, such as World Bank Open Data or United Nations Population Prospects do not provide evidence on global estimates of disability prevalence, hence the information is available separately per country with significant discrepancies between them [2].

The most recent analyses report as much as 1 billion (13%) people worldwide experiencing some kind of disability [3]. It is rough estimation without further disaggregation of this population. Relatively high number of persons with disabilities pose challenges for policy makers at different levels (global, state, regional and local) to address proper actions towards this population requiring recognition and support. The basic document creating a new approach towards persons with disabilities is the UN Convention on the Rights of Persons with Disabilities (UNCRPD)^1^ that set several guidelines^2^ especially with regard to public policy. The UNCRPD approach is based on right of persons with disabilities to enjoy life equally with the rest of the population*.*

This message coming from the UNCRPD has two important implications. The first one concerns the concept of measuring the final impact of implementing the Convention, in other words how to conceptualize fulfillment of rights to enjoy life. The literature provides useful concepts to measure life enjoyment, including quality of life [4–13], wellbeing [14], life satisfaction [15–17], happiness [18] or human flourishing [19, 20].

In this study we have decided to use the quality of life concept. The main reason is that QoL has a multidimensional character and recalls to both subjective (reflecting individual perspective of persons with disabilities) and objective (reflecting objective conditions and availability of resources or support for persons with disabilities) measures [21–25]. The guidelines proposed by the Convention refer to both direct monitoring of system solutions that are supposed to ensure equal rights in all life spheres for population with disabilities [21] as well as subjective perspective of a person with disabilities. The QoL concept capture those two perspectives [26]. The second implication of applying QoL refers to the need of comparisons with population without disabilities as a reference point of equal rights.

The Convention itself obliges countries that signed the document to monitor its implementation (article 31) by collecting appropriate information, including statistical and research data. Voluntary working group - The Washington Group on Disability Statistics - has been set up to define measurement standards and monitor the Convention [3]. These activities did not bring the solutions for gaps in disability data, though.

In this paper we propose to monitor the fulfilment of UNCRPD in an indirect way using the quality of life concept. In its conceptual part the proposed tool is based on the Sen’s capability approach [27–32]. Technically, the operationalisation of the QoL measurement is carried out using a special case of the structural equation model (SEM): namely, the multi-indicators and multiple causes model (MIMIC). This model factors in the different ways of functioning of individuals that lead to different capability levels (quality of life) in different countries. Moreover, it allows to identify individuals’ personal, social, and environ-mental characteristics that strengthen or weaken their QoL.

In the empirical part we implemented the proposed tool using the EU-SILC 2018 database for Poland. The occurrence of disability in Poland is at an average for European Union with 25.8% of women (UE – 26.1%) and 22.7% of men (EU – 21.8%) reporting long-standing limitations in usual activities due to health problems [33]. Poland signed the UNCRPD convention in 2006, and is facing a policy change towards persons with disabilities adopting regulations implementing the UNCRPD [34]. Establishing a monitoring tool for the UNCRPD implementation is important not only for Poland, but also for other countries. A proposed tool can be also used to monitor other policy frameworks towards persons with disabilities.

This paper makes two contributions – methodological and cognitive. We propose a tool aimed at monitoring implementation of the UNCRPD or other policy frameworks. The cognitive contribution of the paper is our comparative analysis of the QoL among the population of Poland with respect to disability status in 2018. In the empirical part of the study, we aim to provide answers to the following questions:

- What is a difference in average QoL of people with disabilities compared to the rest of the population,
- Which determining factors contribute the most to the lower QoL among people with disabilities?

## Background

The concept of QoL appeared in the public discourse in 1960’s as an alternative to prevailing social development goals, which were at that time defined as an improvement in material living conditions [35]. Although the term is commonly used, there is no single, universally accepted definition of quality of life. The World Health Organization’s definition focuses on individuals’ perceptions of their position in life and the correspondence with their expectations. Other definitions include satisfaction with needs, objective, and subjective evaluations of different domains of life, agency and meaning of life. It is gaining importance in the area of healthcare and, as such, it is identified as an outcome of the efficacy of the treatment [36]. Hence, the concept is multifaceted, multidimensional, ambiguous, and requires a clear definition before beginning the research.

In this article we apply the individual-referenced definition outlined by Schalock et al. [37], in which authors underline that QoL is a multidimensional phenomenon composed of core domains influenced by personal characteristics and environmental factors. The authors claim that core domains are the same for all people, although they may vary individually in relative value and importance.

On general level we can distinguish two approaches in measurement of quality of life. The first one is connected with measuring QoL for the total population or its particular sub-groups (present mostly on sociological, economic and demographic research). The second one is dedicated strictly to population with particular activity limitations, which are usually connected with some kind of disability or disabilities (present mostly in medical and socio-medical research).

In case of the first approach the most complex and precise concept of measurement is provided by the final report of the Sponsorship Group ‘Measuring Progress, Well-being and Sustainable Development’ and Task Force on ‘multidimensional measurement of quality of life’ [38], which refers to recommendations Report on Measurement of Economic Performance and Social Progress [39]. This approach underlines multidimensional character of QoL as well as the necessity to combine both subjective and objective measures.

The second concept of quality of life - related to health or activity limitations - originally have been aimed at physical symptoms or mortality [40, 41], but nowadays, it is widely recognized that quality of life is a goal of all healthcare interventions [42]. In disability research it has been suggested that quality of life and participation should be considered the key outcomes [43, 44]. In this approach, quality of life is also considered as a multidimensional construct, which includes physical, mental and social domains [5], however it is studied for particular groups of persons with disabilities, distinguished by the type of disability or impairment. Within this approach, the measurement of QoL is based on the impact of medical procedures on symptoms and the frequency of complications [6–13]. Subjective assessment of QoL is based on the perception of activity limitation on a person’s psychological, emotional health and social functioning [45].

**Fig. 1:**
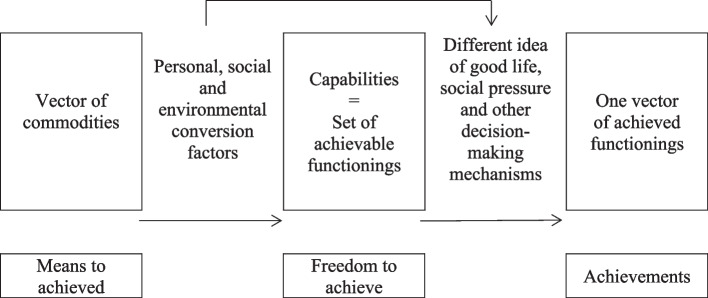
Relationship between commodities, capabilities and functionings in the capability approach. *Source: own study on the basis of* [38]

Sen’s capabilities approach offers a conceptual framework that enables the QoL measurement combining the elements of the two approaches mentioned above [27–32]. It is based on the assumption that commodities themselves are not crucial in achieving a high quality of life. It is their properties that enable achievement of desired lifestyles by individuals. According to Nussbaum and Sen [46], capabilities refer to effective possibilities of realising achievements and fulfilling expectations, whereas functionings, that are the “beings and doings” of a person, refer to realised achievements and fulfilled expectations. Graphical illustration of the relationship between commodities, capabilities and functionings, using the key concepts of the capability approach, is demonstrated in Fig. 1.

The achieved functionings are the result of personal choice to select from the capabilities available and subject to personal preferences, social pressure and other decision-making mechanisms. Moreover, they are constrained by personal, social and environmental characteristics [47, 48]. Due to the above-mentioned theoretical considerations, we decided to use the capability approach to measure QoL for both populations: persons with and without disabilities.

## Methods

### Methodological approach

The proposed methodological approach combines three aspects: (1) measuring QoL within the capabilities approach conceptual framework; (2) the guidelines of the European Statistical System on indicating domains in which the QoL should be measured [38]; (3) the UNCRPD monitoring requirements by focusing on those domains which are explicitly pointed by the UNCRPD.

Selecting domains for multidimensional socio-economic concept can be based on five criteria: existing data or convention, assumptions, public consensus, ongoing deliberative participatory process, and empirical evidence regarding people’s values [49]. Following this approach, we decided to choose quality of life domains based on three criteria: 1. making assumptions based on a theory (capabilities approach); 2. drawing on an existing list that was generated by consensus (the Eurostat Guidelines and the UNCRPD); 3. processes: using existing data (as described further in this section).

Taking all above into account we focus in this study on the following QoL domains:

- material living conditions (art. 28 of the UNCRPD),
- productive or main activity (art. 27 of the UNCRPD),
- health (art. 25 of the UNCRPD),
- education (art. 24 of the UNCRPD),
- leisure and social interactions (art. 30 of the UNCRPD),
- economic and physical safety (art. 14, 16, 19 of the UNCRPD).

The UNCRPD can be considered as a right-based framework to persons with disabilities in different aspects of functionings, representing various life domains. The Convention emphasise the right to self-determination and empowerment as a core issue [50]. Taking this into account policy towards persons with disabilities should be focused on creating possibilities understood as a set of solutions to be chosen by a person with disabilities and tailored to his or her needs. Therefore this approach is consistent with capabilities approach where a right to choose desired lifestyle from a set of achievable functionings is essential.

The UNCRPD assumes the equality between persons with and without disabilities, which suggests, that designing a monitoring tool only for persons with disabilities do not have a comparability advantage [51]. Hence we apply the same measurement process to persons with and without disabilities. Two separate measurement models were estimated – for persons with disabilities and without them. Taking this approach we assume that persons with disabilities can attribute different values to particular life domains than the rest of the population. Finally, the standardisation procedure (described below) makes comparisons between those two populations possible.

In order to operationalize the measurement of quality of life within the framework of capabilities approach (Sen), this paper proposes to apply a MIMIC model that was formulated by Hauser and Goldberger [52], and then popularized by Jöreskog & Goldberger [53], who presented its detailed assumptions as a special case of the structural equation model (SEM) [54, 55]. Krishnakumar [56] pointed at the SEM approach as the most suitable tool for estimating latent capabilities. The MIMIC model allows to explain the level of individual’s quality of life and to assess the impact of external determinants (individual’s personal, social, and environmental characteristics) on latent capabilities. Although, the method does not allow for the identification of causal relations it can shed light on dependencies between determinants and QoL of people with and without disabilities.

The operationalization of measurement of quality of life under a MIMIC model is based on the assumption, that the freedom of individual choice in capabilities is represented by an unobservable latent variable, which can be estimated based on two sets of observable variables:

- the reflective part of the model (measurement sub-model), constructed using a set of selected basic indicators of quality of life (quality of life symptoms), and these variables can be interpreted as realised functionings, potentially reflecting quality of life.
- the formative part of the model (structural sub-model), constructed on the basis of the individuals’ personal, social and environmental exogenous characteristics, which are interpreted as the conversion factors that strengthen or weaken the capabilities and influence the process of transformation of available resources into achieved functioning [56].

**Fig. 2:**
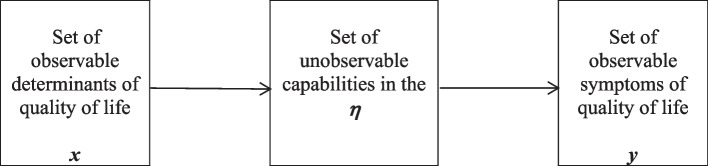
MIMIC model for quality of life as part of the capability approach. Source: own study

The starting point for building the MIMIC model is to define symptoms and determinants of quality of life. The relationships between latent variables (capabilities) and observable variables (quality of life symptoms and determinants) are presented in Fig. 2.

Persons with and without disabilities can, due to different individual resources, possibilities as well as preferences, maximize their quality of life (realise achievements) in various ways. Furthermore, their personal, social, and environmental characteristics can strengthen or weaken their capabilities in different ways. Considering these differences, we estimated two separate models (for persons with and without disabilities), transforming all symptoms and determinants into stimulants (the higher the variable value, the higher the quality of life). In both models theoretical values of latent capabilities were estimated for every individual. These values can be interpreted as QoL in domains. In order to compare those values across models we propose a standardization procedure:

$${QoL}_{S;i;j}=\frac{QoL_{i;j}-{\min}_i\left({QoL}_{i;j}\right)}{\max_i\left({QoL}_{i;j}\right)-\textrm{mi}{\textrm{n}}_i\left({QoL}_{i;j}\right)},$$

where:

*QoL*_*S*; *i*; *j*_ – is a standardized value of QoL of i-th individual in the j-th domain,

*QoL*_*i*; *j*_ – is an estimated value of QoL of i-th individual in the j-th domain.

The minimum value of latent variable reflecting quality of life $({\min}_{i}({QoL}_{i;j}))$ is estimated for an artificial person who had the lowest values of all symptoms and determinants of QoL, and the maximum value $({\max}_{i}({QoL}_{i;j}))$ is estimated for an artificial individual with the highest achievable values of all symptoms and determinants of QoL. Thus, the standardized values of the latent variable allow for comparative analysis of QoL of groups of persons with and without disabilities, while taking into consideration that they can maximise their QoL in a distinct way, and that their personal, social, and environmental characteristics can strengthen or weaken their capabilities differently.

Having the QoL scores estimated for each domain, it is possible to aggregate them into a single overall QoL score. We used factor analysis as a method of aggregation as it serves two purposes – first it allows only information that is shared by at least two basic dimensions, secondly it allows for the reduction of redundant information that is common among dimensions of QoL. By using factor analysis we implicitly assume that the QoL of life is a broad underlying latent variable that is manifested by its numerous symptoms (basic indicators) which are arbitrary grouped into dimensions of quality of life. FA allows for the indirect identification of the said latent variable.

The identification for persons with disabilities was based on the commonly used measure of disability – Global Activity Limitation Indicator (GALI) [57]. This question has three categories: 1) strongly limited in daily activities, 2) limited, but not strongly, 3) not limited at all. All persons who were at least limited (1 and 2) in their activities are defined as those with disabilities, and others – without disabilities.

**Fig. 3:**
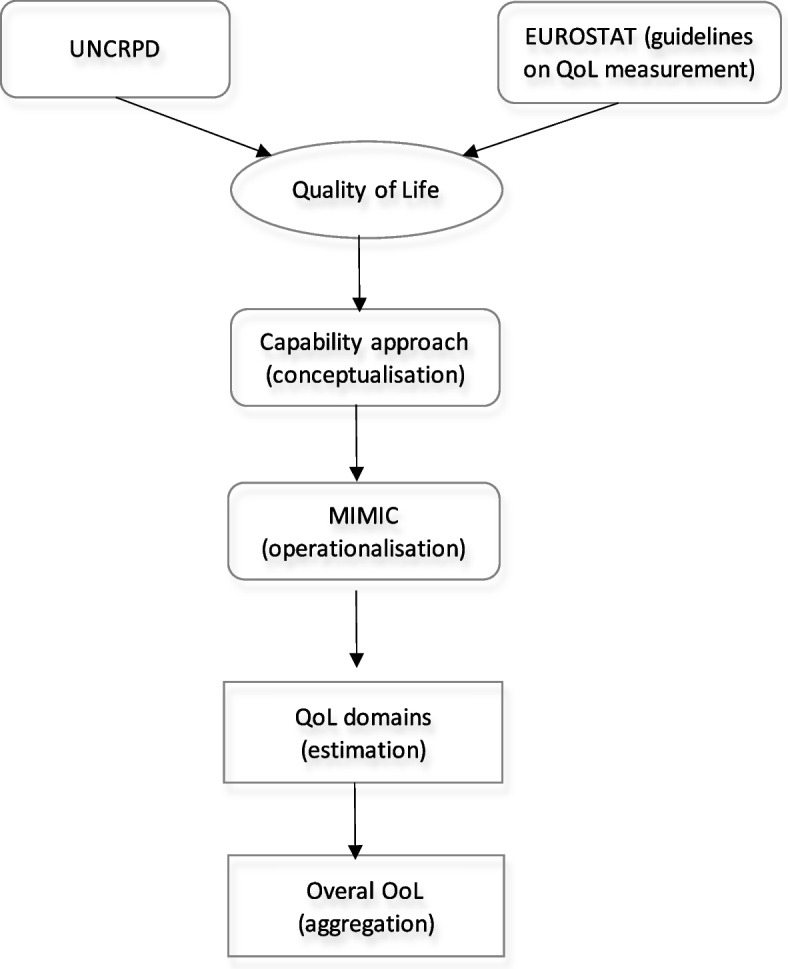
Methodological concept of the paper. Source: own study

To sum up, the methodological concept of the paper is presented in Fig. 3.

### Data

We use data from EU-SILC survey conducted in 2018 in Poland. All information on the access to the EU-SILC dataset can be found on the URL (https://ec.europa.eu/eurostat/web/microdata/european-union-statistics-on-income-and-living-conditions) – access November 2020. This survey is carried out under EU resolution on a sample representative for the Polish population aged 15 years and over. Total sample size for persons with disabilities was 7666 (59.0% of women) and for persons without disabilities was 25,714 (51.5% of women). Estimated frequencies for basis socio-economic variables for population with disabilities, are presented in the Table 1.

**Table 1:** Estimated frequencies for persons with disabilities in Poland, 2018, EU-SILC database

Characteristics	Persons with disabilities		Confidence interval	
	n	%	min	max
**Sex**				
Man	3145	22.17	21.31	23.04
Woman	4521	25.43	24.63	26.36
**Age**				
up to 24 years	151	5.23	4.24	6.22
25–34	255	7.69	6.63	8.75
35–44	430	10.05	8.97	11.14
45–54	787	18.07	16.66	19.47
55–64	1885	31.9	30.39	33.41
65–74	2093	39.91	38.27	41.56
75 years and over	2065	62.82	60.78	64.85
**Level of urbanisation**				
Rural area	2206	24.78	23.89	25.67
Intermediate area	1771	24.12	22.9	25.34
Densely populated area	3689	22.85	21.83	23.88
**Relationship status**				
Single	3013	28.78	27.72	29.94
Living with a partner	4653	21.35	20.65	22.05
**Household size**				
1 person	1525	40.86	38.99	42.73
2 persons	3273	31.18	30.07	32.29
3 persons	1334	21.37	20.18	22.62
4 persons	743	14.47	13.27	15.65
5 and more persons	791	17.65	16.36	18.95
**Equivalised income quartiles**				
bottom	2829	32.59	31.37	33.80
2 nd	2206	25.75	24.59	26.96
3 rd	1584	21.91	20.72	23.10
top	1047	15.24	14.18	16.30
**Education level**				
Primary and below	2259	39.19	37.61	40.77
Vocational	2639	26.83	25.72	27.94
Secondary	1711	21.02	19.85	22.19
Tertiary	794	13.57	12.62	14.51
**Employment status**				
Working	1433	9.88	9.27	10.48
Not working	6233	38.92	37.98	39.87
**Self-rated health**				
Very good	49	1.4	0.93	1.88
Good	651	4.99	4.53	5.46
Fair	3515	39.51	38.24	40.78
Bad	2695	79.26	77.53	80.98
Very bad	754	91.86	89.27	94.44
Total	6615	23.96	23.37	24.54

Source: own calculations

## Results

The description of results refers to life domains. Presentation of the results in each life domain is divided in two parts: the measurement part (in brackets we provide factor loadings) and structural parts (where we point the significance of each determinant in particular domains). In the text we present the path diagrams which can serve as graphical illustration of the results. All detailed results are presented in the Additional file 2.

### QoL scores and distributions

The average quality of life scores are presented in Table 2. The biggest difference in favor of persons without disabilities is observed for health (0.32) and for productive and main activity (0.26) domains. It is connected with health status of persons with disabilities, being influenced by their older age structure, which consists mostly of persons over 55 years old. Health problems and older age result in lower activity of persons with disabilities, especially on the labour market. For material conditions and economic security and physical safety there was a moderate difference recorded. This can be attributed to the stable sources on income (although not very high), connected with pension or disability allowances. For the leisure and social relations domain there is almost no difference. Taking all this into account after aggregation persons without disabilities enjoy on average higher overall QoL than persons without disabilities (0.65 vs 0.50).

**Table 2:** QoL scores by domains in Poland, 2018, EU-SILC database

QoL domain	Persons with disabilities	Persons without disabilities	Difference
Overall QoL	0.50	0.65	0.15***
Material conditions	0.47	0.59	0.12***
Productive and main activity	0.23	0.49	0.26***
Economic security and physical safety	0.57	0.70	0.13***
Health	0.39	0.71	0.32***
Leisure and social relations	0.62	0.63	0,01***

*** Results statistically significant, *p* = 0.000

Source: own calculations

**Fig. 4:**
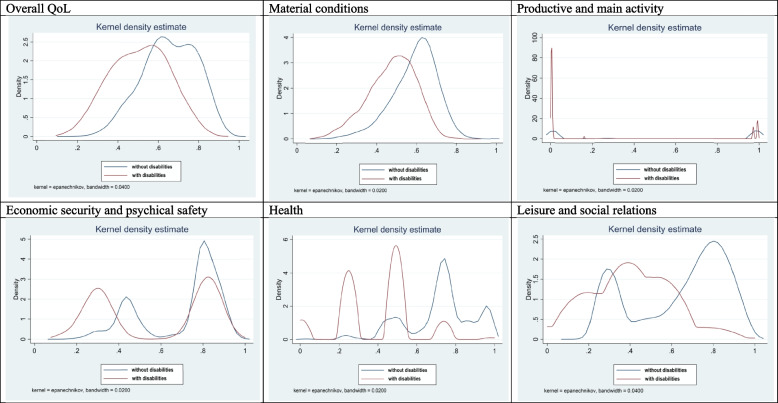
Density functions of the QoL in Poland, 2018, EU-SILC database. Source: own calculations

Information on distribution of the QoL in each life domains is presented in Fig. 4.

For material living conditions the shape of the distribution is similar for both groups. Persons with disabilities experience on average lower values of QoL. Moreover, the distribution for persons with disabilities is shifted to the left. The distribution for the population with disabilities is skewed to the right, whereas for persons without disabilities slightly to the left.

For productivity and other activities dimension we can observe different shapes of the analyzed density functions for both compared populations mainly due to high inactivity of population with disabilities. For population with disabilities, the quality of life in this dimension is strongly skewed to the right – majority of persons take values lower than the mean in this dimension. Whereas for population without disability the distributions is skewed to the right and much more flattened.

In the economic security and personal safety both compared distributions follow similar pattern (bimodal). Distribution for persons with disabilities is shifted to the left. Higher density for persons without disabilities is especially visible at the top end of the distribution. This insecurity of population with disabilities reflects limited earning possibilities and dependence from the state support.

Density functions in the dimension of leisure and social activity follow different patterns for analysed populations. For population with disabilities the distributions is skewed to the right, whereas population without disability to the left. It translates into a significant difference between both population across the whole distribution in this dimension. It reflects both personal and social limitations of persons with disabilities.

We observe that density functions in health dimension for both compared population is tri-modal. For population with disabilities the distribution is shifted to the left. Probably the modes can be attributed to stages in which there is a significant shift in health status. It is striking that the estimated score for persons without disabilities at the top end of the distribution is significantly higher than for persons with disabilities. Distribution for population with disabilities is skewed to the right, whereas for persons without disabilities to the left.

Finally, the distributions of overall QoL for both populations are similar, however, persons with disabilities experience on average lower values of QoL. Moreover, the distribution for persons with disabilities is more dispersed. Population with disabilities is less homogenous in terms of overall QoL and its distribution is more skewed toward left tail, which suggests that disability might be a factor which not only negatively affects quality of life on average, but also can be associated with outlying observations in the left tail of the distribution, which correspond to persons with severely decreased quality of life.

Summarizing, we observe, that in most of the domains the distribution’s right tail is significantly heavier for persons without disabilities (see Fig. 4). This suggests, that lower average quality of life of persons with disabilities is a result of a lower share of those who experience high and very high QoL. The share of persons with low QoL is similar in both groups, however, persons with disabilities relatively rarely achieve high levels of QoL.

### Quality of life in different domains

Quality of Life in each domain is reflected by symptoms measured directly with the use of a questionnaire. Number of variables as symptoms varies from three to seven in every domain. Full list of variables used as symptoms of QoL is presented in Table 3.

**Table 3:** List of variables used as symptoms for each life quality domain

Domains	Indicators
1. Material conditions	1.1. Equivalent income 1.2. Being impoverished 1.3. Satisfaction with financial situation 1.4. Severe material deprivation rate 1.5. Ability to make ends meet 1.6. Poor housing conditions 1.7. Overcrowded dwelling
2. Productivity or other main activity (Productivity)	2.1. Economic activity 2.2. Worked last week 2.3. Long-term unemployed 2.4. People living in households with very low work intensity 2.5. Low-wage earners 2.6. Job satisfaction
3. Health	3.1. Self-perceived health 3.2. Long-term illness 3.3. Unmet needs for medical care
4. Leisure and social interactions (Leisure-Social-Interactions)	5.1. Frequency of getting together with friends (social meetings) 5. 2. Leisure activities 5. 3. Financial obstacles to leisure participation 5.4. Help from others (having someone to rely on in case of need) 5.5. Loneliness 5.6. Satisfaction with relations 5.8 Satisfaction with leisure
6. Economic security and physical safety (Security-Safety)	6.1. Ability to face unexpected financial expenses 6.2. Persons in arrears 6.3 Lost job 6.4. Feeling of safety (people feeling safe when walking alone in their area after dark)

Source: own study

However by conducting separate estimations for persons with and without disabilities, we allow on different latent structures between those populations, because persons with and without can enjoy life in different ways. Results of the measurement part of the estimations are presented in Table 4.

**Table 4:** Estimations of the measurement part in Poland, 2018, EU-SILC database

**Material living conditions**									
**Persons without disabilities**					**Persons with disabilities**				
**Variable**	**Factor loading**	**Std.Err.**	**z**	**P > |z|**	**Variable**	**Factor loading**	**Std.Err.**	**z**	**P > |z|**
Equivalent income	1	(constrained)			Equivalent income	1	(constrained)		
Constant term	0.35329	0.01897	18.62	0	Constant term	0.13064	0.03023	4.32	0.000
Impoverished	−0.70629	0.01618	−43.66	0	Impoverished	−1.27950	0.03542	−36.13	0.000
Constant term	−0.22334	0.01445	−15.45	0	Constant term	−0.29055	0.03948	−7.36	0.000
Income satisfaction	1.08951	0.01962	55.53	0	Income satisfaction	1.53749	0.03711	41.43	0.000
Constant term	0.42850	0.01975	21.7	0	Constant term	0.24532	0.04542	5.4	0.000
Material deprivation	−1.17496	0.02033	−57.79	0	Material deprivation	−1.84262	0.04217	−43.7	0.000
Constant term	−0.42318	0.02086	−20.29	0	Constant term	−0.31311	0.05348	−5.85	0.000
Making ends meet	1.25664	0.02145	58.57	0	Making ends meet	1.67861	0.03837	43.75	0.000
Constant term	0.43747	0.02229	19.63	0	Constant term	0.31691	0.04873	6.5	0.000
Poor housing conditions	−0.40557	0.01496	−27.11	0	Poor housing conditions	−0.70389	0.03394	−20.74	0.000
Constant term	−0.16726	0.01075	−15.56	0	Constant term	−0.15016	0.02691	−5.58	0.000
Over-crowded dwelling	−0.40740	0.01546	−26.35	0	Over-crowded dwelling	−0.44083	0.02726	−16.17	0.000
Constant term	−0.21327	0.01010	−19.39	0	Constant term	−0.36566	0.01953	−18.73	0.000
**Productive and main activity**									
**Persons without disabilities**					**Persons with disabilities**				
**Variable**	**Factor loading**	**Std.Err.**	**z**	**P > |z|**	**Variable**	**Factor loading**	**Std.Err.**	**z**	**P > |z|**
Economic activity	1	(constrained)			Economic activity	1	(constrained)		
Constant term	0.15439	0.00640	24.17	0	Constant term	0.29457	0.01730	0	0.261
Worked last week	1.03559	0.00291	356.43	0	Worked last week	1.01385	0.00430	0	1.005
Constant term	0.14425	0.00643	22.43	0	Constant term	0.30233	0.01714	0	0.269
Long-term unemployed	−0.10772	0.00733	−14.7	0	Long-term unemployed	−0.03595	0.01464	0.014	−0.065
Constant term	0.04873	0.00698	6.98	0	Constant term	−0.01382	0.01444	0.339	−0.042
Low work intensity	−0.27743	0.00626	−44.36	0	Low work intensity	−0.01570	0.01443	0.276	−0.044
Constant term	0.21055	0.00652	32.3	0	Constant term	−0.02361	0.01452	0.104	−0.052
Low income earners	0.47099	0.00633	74.43	0	Low income earners	0.77491	0.00797	0	0.759
Constant term	0.11607	0.00695	16.69	0	Constant term	0.28878	0.01522	0	0.259
Job satisfaction	0.01860	0.00650	2.86	0.004	Job satisfaction	−0.15061	0.01210	0	−0.174
Constant term	0.01519	0.00659	2.31	0.021	Constant term	−0.15132	0.01253	0	−0.176
**Health**									
**Persons without disabilities**					**Persons with disabilities**				
**Variable**	**Factor loading**	**Std.Err.**	**z**	**P > |z|**	**Variable**	**Factor loading**	**Std.Err.**	**z**	**P > |z|**
Health self-assessment	1	(constrained)			Health self-assessment	1	(constrained)		
Constant term	0.48047	0.00491	97.9	0	Constant term	−0.75075	0.01686	−44.54	0.000
Long-term illness	−0.55564	0.00878	−63.25	0	Long-term illness	−0.12518	0.02288	−5.47	0.000
Constant term	−0.27368	0.00407	−67.31	0	Constant term	1.38315	0.00833	166.08	0.000
Unmet medical needs	−0.20113	0.00759	−26.49	0					
Constant term	−0.04291	0.00497	−8.64	0					
**Social relations and leisure**									
**Persons without disabilities**					**Persons with disabilities**				
**Variable**	**Factor loading**	**Std.Err.**	**z**	**P > |z|**	**Variable**	**Factor loading**	**Std.Err.**	**z**	**P > |z|**
Social meetings	1	(constrained)			Social meetings	1	(constrained)		
Constant term	0.32965	0.01894	17.4	0	Constant term	0.68039	0.03944	17.25	0.000
Leisure activities	0.77766	0.00865	89.87	0	Leisure activities	0.74390	0.02714	27.41	0.000
Constant term	0.32894	0.01553	21.18	0	Constant term	0.26086	0.03294	7.92	0.000
Non-material help	0.64767	0.01038	62.41	0	Non-material help	0.42948	0.03124	13.75	0.000
Constant term	0.12984	0.01350	9.59	0	Constant term	0.56087	0.02553	21.97	0.000
Loneliness	0.55936	0.01705	32.8	0	Loneliness	1.19503	0.05019	23.81	0.000
Constant term	0.11607	0.01256	9.24	0	Constant term	0.50258	0.04719	10.65	0.000
Satisfaction with relations	0.63592	0.01710	37.2	0	Satisfaction with relations	1.20900	0.04731	25.55	0.000
Constant term	0.06936	0.01385	5.01	0	Constant term	0.62890	0.04465	14.08	0.000
Satisfaction with leisure	0.21599	0.01721	12.55	0	Satisfaction with leisure	0.59130	0.03573	16.55	0.000
Constant term	−0.04373	0.00907	−4.82	0	Constant term	0.50568	0.03019	16.75	0.000
**Economic security and physical safety**									
**Persons without disabilities**					**Persons with disabilities**				
**Variable**	**Factor loading**	**Std.Err.**	**z**	**P > |z|**	**Variable**	**Factor loading**	**Std.Err.**	**z**	**P > |z|**
Unexpected expenses	1	(constrained)			Unexpected expenses	1	(constrained)		
Constant term	0.46024	0.02204	20.88	0	Constant term	0.15947	0.05100	3.13	0.002
Persons in arrears	−0.39831	0.02390	−16.67	0	Persons in arrears	−0.35385	0.03637	9.73	0.000
Constant term	−0.16800	0.01374	−12.23	0	Constant term	−0.09484	0.02522	3.76	0.000
Lost job	−0.10891	0.01395	−7.81	0	Lost job	−0.03372	0.01413	2.39	0.017
Constant term	−0.03238	0.00868	−3.73	0	Constant term	−0.04297	0.01165	3.69	0.000
Safety feeling	0.12958	0.01511	8.58	0	Safety feeling	0.09572	0.01979	4.84	0.000
Constant term	0.05749	0.00884	6.5	0	Constant term	0.01242	0.01541	0.81	0.42

Source: own study

In case of material conditions domain crucial for QoL for persons with disabilities is material deprivation and subjective assessment of material conditions rather than objective indicators. In case of persons without disabilities both subjective and objective indicators are crucial of QoL in this domain.

The QoL in productive and main activity domain is reflected first of all by indicators connected with labour market participation: economic activity and working last week both for persons with and without disabilities. Such indicators as low income job, job satisfaction, low work intensity or long-term unemployment reflects QoL score in that domain in small extend for both analysed populations.

The QoL in the domain of economic security and physical safety is mostly reflected in the ability to face unexpected expenses, both for analyzed populations. Also being indebted reflects the QoL in this domain for both types of populations, although in weaker way.

For persons with and without disabilities the QoL in the health domain is reflected mainly in the health self-assessment. For persons without disabilities crucial role can be attributed also to long term illness, whereas for persons with disabilities this indicator reflects QoL in this domain in much smaller degree. Also unmet medical needs can be considered as a symptom of the QoL for persons without disabilities in this domain, but of a much weaker strength.

In the domain of leisure and social relations crucial role for persons without disabilities can be assigned to the frequency of meeting together with friends. Other symptoms, such as leisure activities, possibility to receive non-material help from others, loneliness, satisfaction with relations and satisfaction with leisure reflect the QoL in this domain to smaller extent. Whereas for persons with disabilities the most stronger symptoms of the QoL in this domain are loneliness and satisfaction with relations, followed by the frequency of meeting together with friends. Leisure activities and satisfaction with leisure reflects the QoL for persons with disabilities in a smaller degree. The least symptomatic nature for persons with disabilities in this domain can be assigned to possibilities to receive non material help for others.

### Determinants of quality of life

Interesting part of the results are the patterns of the influence of the determinants of the QoL score across domains (see Table 5).

**Table 5:** Estimations of the structural part in Poland, 2018, EU-SILC database

**Material living conditions**									
**Persons without disabilities**					**Persons with disabilities**				
**Variable**	**Coefficient**	**Std.Err.**	**z**	**P > |z|**	**Variable**	**Coefficient**	**Std.Err.**	**z**	**P > |z|**
Age	0.09854	0.00888	11.1	0	Age	0.17692	0.01264	14	0.000
Gender	−0.06026	0.00516	−11.67	0	Gender	−0.01216	0.00660	−1.84	0.065
Having a partner	0.14497	0.00609	23.8	0	Having a partner	0.12242	0.00726	16.87	0.000
Urbanisation degree	0.02865	0.00515	5.56	0	Urbanisation degree	0.04454	0.00660	6.75	0.000
Vocational education	0.00350	0.00628	0.56	0.577	Vocational education	0.02941	0.00672	4.38	0.000
Secondary education	0.09007	0.00609	14.8	0	Secondary education	0.08750	0.00695	12.58	0.000
Tertiary education	0.17838	0.00627	28.44	0	Tertiary education	0.15925	0.00841	18.94	0.000
Employ	−0.01150	0.00438	−2.62	0.009	Employ	−0.01121	0.00531	−2.11	0.035
Household size	−0.00294	0.00629	−0.47	0.64	Household size	0.08496	0.00892	9.53	0.000
Healts self-assessment	−0.19714	0.00806	−24.47	0	Health self-assessment	−0.15178	0.00835	−18.17	0.000
**Productive and main activity**									
**Persons without disabilities**					**Persons with disabilities**				
**Variable**	**Coefficient**	**Std.Err.**	**z**	**P > |z|**	**Variable**	**Coefficient**	**Std.Err.**	**z**	**P > |z|**
Age	−0.32706	0.00898	−36.43	P > z	Age	−0.51489	0.01491	−34.54	0.000
Gender	−0.10196	0.00512	−19.92	0	Gender	−0.02300	0.00869	−2.65	0.008
Having a partner	0.25240	0.00614	41.08	0	Having a partner	0.08509	0.00903	9.42	0.000
Urbanisation degree	−0.03549	0.00530	−6.7	0	Urbanisation degree	−0.00830	0.00869	−0.95	0.34
Vocational education	0.32602	0.00638	51.08	0	Vocational education	0.05161	0.00887	5.82	0.000
Secondary education	0.26893	0.00600	44.81	0	Secondary education	0.04521	0.00918	4.92	0.000
Tertiary education	0.37613	0.00592	63.56	0	Tertiary education	0.14812	0.01074	13.79	0.000
Household size	−0.01516	0.00604	−2.51	0	Household size	0.02944	0.01036	2.84	0.005
Healts self-assessment	0.07078	0.00803	8.82	0.012	Health self-assessment	0.13959	0.01022	13.65	0.000
**Health**									
**Persons without disabilities**					**Persons with disabilities**				
**Variable**	**Coefficient**	**Std.Err.**	**z**	**P > |z|**	**Variable**	**Coefficient**	**Std.Err.**	**z**	**P > |z|**
Age	−0.48655	0.00680	−71.57	0	Age	−0.31752	0.01592	−19.94	0.000
Gender	−0.04813	0.00412	−11.68	0	Gender	0.03733	0.00973	3.84	0.000
Having a partner	−0.03733	0.00497	−7.51	0	Having a partner	0.06818	0.01008	6.77	0.000
Urbanisation degree	0.00629	0.00432	1.46	0.145	Urbanisation degree	0.01084	0.00973	1.11	0.265
Vocational education	−0.03754	0.00514	−7.3	0	Vocational education	0.02309	0.00994	2.32	0.02
Secondary education	−0.00044	0.00479	−0.09	0.927	Secondary education	0.06631	0.01033	6.42	0.000
Tertiary education	0.04358	0.00463	9.41	0	Tertiary education	0.11918	0.01194	9.98	0.000
Household size	0.03540	0.00500	7.08	0	Household size	−0.00630	0.01163	−0.54	0.588
**Social relations and leisure**									
**Persons without disabilities**					**Persons with disabilities**				
**Variable**	**Coefficient**	**Std.Err.**	**z**	**P > |z|**	**Variable**	**Coefficient**	**Std.Err.**	**z**	**P > |z|**
Age	−0.08054	0.00829	−9.72	0	Age	−0.02594	0.01411	−1.84	0.066
Gender	0.07691	0.00461	16.7	0	Gender	0.04381	0.00803	5.46	0.000
Having a partner	0.22587	0.00554	40.76	0	Having a partner	0.17972	0.00895	20.09	0.000
Urbanisation degree	−0.01500	0.00471	−3.19	0.001	Urbanisation degree	0.03037	0.00782	3.88	0.000
Vocational education	0.25478	0.00574	44.42	0	Vocational education	0.04879	0.00793	6.15	0.000
Secondary education	0.30887	0.00552	55.95	0	Secondary education	0.07832	0.00831	9.42	0.000
Tertiary education	0.30268	0.00543	55.7	0	Tertiary education	0.11756	0.00997	11.79	0.000
Employ	0.04139	0.00441	9.38	0	Employ	0.00967	0.00620	1.56	0.119
Household size	−0.11338	0.00537	−21.12	0	Household size	−0.02176	0.00998	−2.18	0.029
Healts self-assessment	−0.19592	0.00832	−23.55	0	Health self-assessment	−0.22795	0.01075	−21.2	0.000
**Economic security and physical safety**									
**Persons without disabilities**					**Persons with disabilities**				
**Variable**	**Coefficient**	**Std.Err.**	**z**	**P > |z|**	**Variable**	**Coefficient**	**Std.Err.**	**z**	**P > |z|**
Age	0.16006	0.01045	15.31	0	Age	0.26405	0.02075	12.73	0.000
Gender	−0.04608	0.00582	−7.91	0	Gender	−0.04031	0.01183	−3.41	0.001
Having a partner	0.06830	0.00702	9.74	0	Having a partner	0.12497	0.01225	10.2	0.000
Urbanisation degree	−0.00185	0.00633	−0.29	0.77	Urbanisation degree	0.03887	0.01222	3.18	0.001
Vocational education	−0.02883	0.00710	−4.06	0	Vocational education	0.02324	0.01203	1.93	0.053
Secondary education	0.06523	0.00667	9.78	0	Secondary education	0.12263	0.01249	9.82	0.000
Tertiary education	0.14412	0.00664	21.72	0	Tertiary education	0.21891	0.01470	14.89	0.000
Employed	0.02936	0.00572	5.13	0	Employed	0.02454	0.00948	2.59	0.01
Household size	0.09928	0.00692	14.36	0	Household size	0.21837	0.01445	15.11	0.000
Health self-assessment	−0.20329	0.01021	−19.91	0	Health self-assessment	−0.16711	0.01439	−11.62	0.000

Source: own study

Being a men positively influence QoL in material living conditions, productive and main activity, economic security and physical safety and in health domain, but for health only in case of persons without disabilities. Being a women favors QoL in leisure and social relations domain and in case of persons with disabilities also in health domain. So when it comes to domains connected with economic and material situation being a men favors the QoL score, whereas being a women increases the QoL scores in social relations and leisure.

The diversified impact of age on the QoL was observed. For material living conditions and economic security and physical safety domain we can observe a positive impact of age on the QoL, stronger for persons with disabilities than for persons without disabilities. Whereas for productive and main activity, health and social relations the impact is opposite for both analyzed populations.

Household situation is also crucial for the QoL in different domains. Generally possessing a partner positively influence the QoL in all domains, with the exception of health domain for persons without disabilities. Moreover, for persons with disabilities bigger households facilitate the QoL in material living conditions, productive and main activity, economic security and physical safety domain, whereas negative influence of the household size was recorded in domains of health (with a very small effect) and social relations. Presence of other household members in case of persons with disabilities improve material and economic situation by providing additional income sources and enabling more engagement on the labour market leading also to improving the QoL.

The impact of education is important determinant of the QoL score in all domains, for persons with disabilities the pattern of influence for all educational levels across all domains is positive – the higher the level of education the higher the QoL. For persons without disabilities the general impact is also positive (even stronger than for persons with disabilities, although the pattern of influence by different educational levels across domains is more diversified.

In case of urbanization degree the pattern of influence is diversified. For persons with disabilities the less urbanized area of living the higher the QoL in material living conditions, economic security and physical safety, social relations and leisure domain, in the rest of domains the influence was statistically insignificant. In case persons without disabilities living on less urbanized areas favors the QoL in material living conditions and health domains, whereas in productive and main activity and social relations domains the direction of influence is opposite.

## Discussion

The paper made two crucial contributions: methodological and cognitive. The first one is connected with establishing a tool to monitor implementation of the UNCRPD or more generally, to monitor the QoL of persons with disabilities. The proposed method can be calibrated to monitor results of any public policies towards population with disabilities. Thanks to the proposed normalization procedure it allows for comparative analysis across heterogenic population i.e. for cross country comparisons.

The second contribution refers to the comparison of the level of QoL between persons with and without disabilities in Poland, with the application of the above-mentioned tool across different life domains. In this analytical part of the study, we used symptoms and determinants of the QoL in five life domains, indicated by the UNCRPD: material living conditions, productive and main activity, economic security and physical safety, health, leisure and social interactions. Hence, this study presents the possibilities of the analytical to be used as an element of the evidence based policy towards persons with disabilities [58].

The prepared tool combines all core features of QoL measurement present in the literature for the population with and without disabilities [4, 22], these are:

- QoL measurement is composed of the same factors and relationships for all people;
- QoL is experienced when a person’s needs are met and when the individual has the opportunity to pursue life enrichment in major life activity settings;
- QoL is comprised of both subjective and objective components;
- QoL is a multidimensional construct, influenced by individual and environmental factors.

Our approach is based on the assumption, that although persons with disabilities can realize their QoL in different way than persons without disabilities, the measurement process should be similar for both populations if we want to measure equal life enjoyment for those groups. The proposed tool fulfills these requirements. In the literature, the QoL measurement for persons with a particular type of impairment is very subjective and depends on psychological condition, personality, values, norms and attitudes. For example, the same symptoms – difficulties with walking, can mean severe decrease in overall life activity for one person, whereas for the other can be considered and small inconvenience [59]. In this approach the QoL is also divided into domains. The basic domains are: overall approach to health (measured i.e. as self-assessment of health), physical health (measured i.e. as limitations in daily activities, walking limitations), psychological and emotional health (measured as i.e. type and frequency of positive and negative feelings, behavioral and emotional control, concentration, memory), social functioning (measures such as: number of close friends, frequency of meetings with friends. In our approach we diverged from focusing on particular type of impairment, and use domains pointed by Eurostat and present in the UNCRPD.

The tool presented here allows direct comparisons (in scores) of overall and domain QoL for persons with and without disabilities. The applied MIMIC approach enable simultaneous modelling of symptoms and determinants of QoL in each domain.

Not surprisingly, persons without disability experienced higher quality of life as compared to the population with disabilities. Higher QoL scores identified for persons without disabilities in comparison to persons with disabilities were reported in many studies (e.g. [60–62]), but the analysis of distributions of QoL in various life domains bring new insight. Moreover, population with disabilities is more heterogenous, which is in line with the evidence of other researchers, who separately analysed the situation of persons with a particular type of disability or activity limitations [11–13, 63–66]. This suggest the necessity to diversify the public policy towards precisely defined needs of particular groups of persons with disability, and it can be achieved by combining efforts of different parities: persons with disabilities, care providers, service providers, organizations supporting persons with disabilities, local authorities, and other public institutions. Our results can be treated as an argument to create the whole bunch of integrated measures from which policy makers can choose.

The results presented in the structural part of the study are consistent with the literature. Similary to the results of our study (both for persons with and without disabilities), we can find evidence in the literature, that gender influence the QoL, however the impact differs with age, and with income and cultural context (e.g. [67, 68]). The results of the interdependencies between age, gender and QoL depend on the particular measurement tool of the QoL used. In our study, the relationship between age and QoL is diversified across domains for both analyzed groups. Those results reflects the life course perspective and ageing process, that deteriorates health and affect different life activities, highlighting the need to take proper preventive actions [69, 70].

In case of the household situation, the negative impact in social relations domains for persons with disabilities can be associated with the fact, that a need of social contacts is achieved within a household, sililarly to other studies [71, 72]. The positive relation between education and QoL for both analyzed groups, with even stronger impact for persons with disabilities, is also confirmed in other studies, as well as the finding that the pattern of influence by different educational levels across domains is more diversified for persons with disabilities [73, 74]. Other studies also confirm positive relation between living on less urbanized areas and QoL for persons with disabilities, that can be connected with stronger family and community relations in rural areas, where the creation of the support network around persons with disabilities is more frequently encountered [75, 76].

Our research is based on case of Poland, because this country is currently in the process of legislative work implementing the UNCRPD, systemic solutions are being designed to adapt national policy to the new paradigm of support provided to persons with disabilities. In addition, the requirements of the process of deinstitutionalization^3^ of support at the European level make a dignified and independent life of persons with disabilities the main objective of changing public policy towards this group of persons [34]. In this context, both multidimensional approach to QoL as well as the combination of objective and subjective measures of the socio-economic situation of persons with disabilities and their environment are provided by the tool presented in this study.

The empirical analysis does not capture all elements of the UNCRPD, but only those that were measured quantitatively within the dataset used. The tool does not allow for the measurement of elements such as, for example, social attitudes towards persons with disabilities, which may reduce the quality of life of this group. This limitation may become an area for further research into the quality of life of persons with disabilities in Poland. The tool developed in this article enables comparisons between population with and without disabilities in the domains covered by the UNCRPD. Currently, at the European level, proper indicator for measuring QoL for persons with disabilities in comparison to persons without disabilities is missing. For example, Social Scoreboard of European Pillar of Social Rights does not include any indicator covering persons with disabilities (https://composite-indicators.jrc.ec.europa.eu/social-scoreboard/). The proposed tool can fullfil the gap in this area.

The tool itself has also a big potential for comparability on different levels:

- Track changes over time,
- Compare various groups in the same period of time,
- Compare the same group in various countries/regions.

The stability of the EU-SILC database, which was used to calculate the model in this article and its comparability between waves poses the possibility to measure changes over time.^4^ After proper calibration, the tool can be applied to compare the quality of life of persons with disabilities to any other group. Finally, it could be used to compare QoL for persons with disabilities in different countries, with the ability e.g. to prepare the ranking of countries.

The proposed tool can be further tested in other EU countries. The QoL scores calculated in our study should be considered as the long-term outcome of the public policy towards persons with disabilities rather than direct output [77]. The monitoring can be successfully implemented in particular periods of time, depending on the timeliness of data collection.

### Limitations

Our research has few limitations. First is related to data collection. We used survey data and as all information from survey data they are derived from respondent’s answers, which are subjective. It especially applies to the definition of disability, which is based on respondent’s answer and it’s not controlled by the interviewer, hence all classification by disability status we use are also based on respondent’s own assessment and these might vary by individual’s perception.

Second limitation is also related to data collection. EU-SILC respondents’ selection criteria do not include disability status. Therefore, although the survey is representative for total population, it is not representative for the population of persons with disabilities. On the other hand, to our knowledge, a large survey representative for population with disabilities is not available.

Third limitation is connected with the questionnaire design. We used secondary data, and had no influence on how the questions were formulated. It is especially important for quality of life domains and indicators in each domain. Any change of the indicator might slightly change the final results and conclusions, although we believe EU-SILC is a well-recognized tool and the list of indicators was validated Eurostat and researchers.

## Supplementary Information

**Additional file 1.** List of variables used as symptoms for each life quality domain.

**Additional file 2.** Detailed results.

## Data Availability

The dataset analyzed during the current study is available on request via Eurostat following the rules presented on the website, https://ec.europa.eu/eurostat/web/microdata.

## Abbreviations

QoL: Quality of Life
UNCPRD: The UN Convention on the Rights of Persons with Disabilities
EU-SILC: The European Union Statistics on Income and Living Conditions survey
ADL: Activities of Daily Living limitation
MIMIC: Multiple Indicator Multiple Causes model
SD: Standard Deviation,
CV: Coefficient of Variation

## Footnotes

1 Announced in 2006r and signed by 92 signatories (countries).

2 These guidelines are as follows (art. 3 of the CRPD):

- Respect for inherent dignity, individual autonomy including the freedom to make one’s own choices, and independence of persons,
- Non-discrimination,
- Full and effective participation and inclusion in society,
- Respect for difference and acceptance of persons with disabilities as part of human diversity and humanity,
- Equality of opportunity,
- Accessibility,
- Equality between men and women,
- Respect for the evolving capacities of children with disabilities and respect for the right of children with disabilities to preserve their identities.

3 Deinstitutionalisation is defined as a process of transition from organising support based on facility-based solutions to organising support in a community-based way, using the resources of local communities, especially the infrastructure of social services. [Report of the Ad Hoc Expert Group on the Transition from Institutional to Community-based Care handed over to Commissioner Spidla, 2009, https://ec.europa.eu/social/main.jsp?langId=en&catId=89&newsId=614&furtherNews=yes]

4 EU SILC data available on 04.01.2021 at https://ec.europa.eu/eurostat/web/microdata/european-union-statistics-on-income-and-living-conditions
