# Supplementary material for: How to measure multidimensional quality of life of persons with disabilities in public policies - a case of Poland
Source: Arch Public Health. 2022 Nov 10;80:230. doi: 10.1186/s13690-022-00981-5 (PMC9647975; doi:10.1186/s13690-022-00981-5)
Supplement: Supplementary file 1 — Additional file 1. List of variables used as symptoms for each life quality domain. [file 13690_2022_981_MOESM1_ESM.docx]

Annex 1. List of variables used as symptoms for each life quality domain

| Domains | Indicators |
| --- | --- |
| 1. Material conditions | 1.1. Equivalent income  1.2. Being impoverished  1.3. Satisfaction with financial situation  1.4. Severe material deprivation rate  1.5. Ability to make ends meet  1.6. Poor housing conditions  1.7. Overcrowded dwelling |
| 2. Productivity or other main activity  (Productivity) | 2.1. Economic activity  2.2. Worked last week  2.3. Long-term unemployed  2.4. People living in households with very low work intensity  2.5. Low-wage earners  2.6. Job satisfaction |
| 3. Health | 3.1. Self-perceived health  3.2. Long-term illness  3.3. Unmet needs for medical care |
| 4. Leisure and social interactions  (Leisure-Social-Interactions) | 5.1. Frequency of getting together with friends (social meetings)  5. 2. Leisure activities  5. 3. Financial obstacles to leisure participation  5.4. Help from others (having someone to rely on in case of need)  5.5. Loneliness  5.6. Satisfaction with relations  5.8 Satisfaction with leisure |
| 6. Economic security and physical safety  (Security-Safety) | 6.1. Ability to face unexpected financial expenses  6.2. Persons in arrears  6.3 Lost job  6.4. Feeling of safety (people feeling safe when walking alone in their area after dark) |

Source: own study.
